# Transitional care for rheumatic conditions in Europe: current clinical practice and available resources

**DOI:** 10.1186/s12969-017-0179-8

**Published:** 2017-06-09

**Authors:** Daniel Clemente, Leticia Leon, Helen Foster, Loreto Carmona, Kirsten Minden

**Affiliations:** 1 Paediatric Rheumatology Unit, Hospital Infantil Universitario “Niño Jesús”, Madrid, Spain; 2 IDISSC, Hospital Clínico San Carlos; Health Sciences, Universidad Camilo José Cela, Madrid, Spain; 3 Musculoskeletal Research Group, Institute Cellular Medicine, Newcastle University, and Great North Children’s Hospital, Newcastle Hospitals NHS Foundation Trust, Newcastle upon Tyne, UK; 4 Instituto de Salud Musculoesquelética, Madrid, Spain; 5 Department of Rheumatology and Clinical Immunology, and German Rheumatism Research Centre, Charité University Medicine Berlin, a Leibniz Institute, Berlin, Germany

**Keywords:** Transitional care, Rheumatic diseases, Adolescents, Young adults, Chronic disease, Survey method

## Abstract

**Objective:**

To assess European pediatric rheumatology providers’ current clinical practices and resources used in the transition from child-centered to adult-oriented care.

**Methods:**

European pediatric rheumatologists were invited to complete a 17-item anonymized e-survey assessing current transition practices, transition policy awareness, and needs in advance of the publication of EULAR/PReS recommendations on transition.

**Results:**

The response rate was 121/276 (44%), including responses from 115 centers in 22 European Union countries. Although 32/121 (26%) responded that their centers did not offer transition services, the majority (99%) agreed that a formalized process in transitioning patients to adult care is necessary. A minority (<30%) of respondents stated that they have a written transition policy although 46% have an informal transition process. Designated staff to support transitional care were available in a minority of centers: nurse (35%), physiotherapist (15%), psychologist (15%), social worker (8%), and occupational therapist (2%). The existence of a designated team member to coordinate transition was acknowledged in many centers (64% of respondents) although just 36% use a checklist for young people as part of individualized transitional care.

**Conclusion:**

This survey of European pediatric rheumatology providers regarding transitional care practices demonstrates agreement that transitional care is important, and wide variation in current provision of transition services exists.

**Electronic supplementary material:**

The online version of this article (doi:10.1186/s12969-017-0179-8) contains supplementary material, which is available to authorized users.

Juvenile onset rheumatic and musculoskeletal diseases (jRMDs) have high impact on all aspects of the lives of children, young people (YP) and their families. Based on prevalence rates, it is estimated that there are 75,000 children under 16 years old with juvenile idiopathic arthritis (JIA) [1] and about 2500 with systemic lupus erythematosus (SLE) in Europe [2]. These conditions persist into adulthood for many individuals, with continuing disease activity and need for ongoing medication with potential disease-associated morbidity, life-long disability and psychosocial impact [3–5]. During the period of transition, YP with jRMDs have to cope with tremendous physical, emotional and social changes and also with important change in the delivery of clinical care as they move into adult services. Providing transitional care services during this period is necessary to ensure that YP can take control of their health care needs, engage with health care providers and ultimately emerge into adulthood with their optimal function and potential [6, 7]. High-quality, developmentally appropriate healthcare service provision requires the involvement of the YP and the family and also a continuous communication between all persons and provider services involved in transitional care [8].

Whilst the rationale and aims of transitional care in young people with jRMD are clear, organization and delivery of transitional care and what constitutes the ideal service, remains unclear [9, 10]. Previous surveys in the UK, North America and Europe [11–13] have demonstrated variable provision of care and resources, unmet education and training needs for health care providers, and the need for guidance on the process. This state of play facilitated the drive for the EULAR/PReS working party for transitional care to be set up in 2014 with subsequent published recommendations [14]. The purpose of our survey was to assess as a baseline, the European pediatric rheumatology providers’ transition practices and resources in advance of the EULAR/PReS recommendations for transition being widely available.

## Methods

European pediatric rheumatologists were invited to complete an anonymized 17-item e-survey assessing their current transition practice. The questionnaire was developed by the authors (DC, LL, HF, LC, KM) after a systematic literature review and critical appraisal of transitional care programs in rheumatology [10] and two face-to-face meetings of the EULAR/PReS working party for transitional care [14]. Items were included that enquired about current transition practices, agreed key elements of transition programs and available resources (see Additional file 1). For comparability reasons, items 1 to 3 were matched with questions of the North-American CARRA survey by Chira et al. [12]. The survey was distributed by the Coordinating Centre of PRINTO (Pediatric Rheumatology INternational Trials Organisation) via SurveyMonkey© to the PRINTO directors of all PRINTO centers in 25 European Union countries. PRINTO is an international network of academic, clinical centers actively engaged in the research/clinical care of children and adolescents with pediatric rheumatic diseases. One reminder was sent to increase the response rate. The questionnaire included items about the transitional care service, staffing, process and resources. The e-survey was piloted by the authors to optimize clarity and ease of administration online.

The study was conducted in cooperation with PRINTO. We did not collect personal identifiable information, such as telephone numbers, names of individuals or institutions, or addresses. The survey respondents voluntarily participated in the study giving their consent to data collection for the purpose of scientific research.

Descriptive statistics are presented and where possible the results were compared with the North American 2014 survey [12] using proportions z test statistical analysis, assuming independence of the two cohorts and α = 0.01, given the multiple comparisons being made [15, 16]. The responses of rheumatology centers from different levels of care were compared by the chi-squared test. The 99% CI of the difference between surveys is provided.

## Results

### Respondent demographics and characteristics

The link to the survey was e-mailed to pediatric rheumatologists (*n* = 276) from PRINTO centers in 25 European Union countries in April 2016. Of these, 121 responded (44% response rate), representing 115 centers in 22 countries, and thus much of the European Union. Exceptions were Bulgaria, Estonia and Lithuania from whom no responses were received, and Cyprus, Malta and Luxemburg which do not currently have PRINTO centers. The 115 centers who responded correspond to 88% of the PRINTO centers currently listed on the PRINTO-website in those 22 countries. These represent centers with a special interest in research and potentially more likely to have an interest in transition. Most respondents (*n* = 60; 49%) work in a university-affiliated practice, 39% (*n* = 47) in a designated children’s hospital, 4% (*n* = 5) in private practice and 7% (*n* = 8) in other settings.

## Transition policy, staff involved, tools and resources

Most (*n* = 120; 99%) respondents regarded a transition policy as being necessary for good clinical practice. The majority stated that they follow an informal transitional care with only one in four centers having a written transition policy (Table 1). Most rheumatology centers (respondents *n* = 88; 73%) regularly offer transition services for YP with rheumatic diseases, with a wide range of health professionals involved including pediatric and adult rheumatologists and nurses being most common (Table 2). There is a designated staff member who has primary responsibility for coordinating transition process in approximately two thirds of centers (respondents *n* = 77, 64%); the staff member is often a doctor (*n* = 60; 78%) or a nurse specialist (*n* = 14; 18%). A minority (respondents *n* = 9/119; 7.6%) of transition units receive designated funding or reimbursement for their services, with health insurance companies, grants, pharmaceutical companies, or university as sources.

**Table 1:** Current transition policies at the respondents’ units

Regarding transition policies….*	Number	Percent
We have a written transition policy, which we follow most of the time.	29	23.9
We have a written transition policy, but we do not follow it most of the time.	4	3.3
We do not have a written transition policy, but follow a fairly standard, informal procedure in transitioning our patients.	56	46.2
We are working on developing a transition policy, but do not yet have one formalized.	21	17.3
We do not have a transition policy, but are interested in developing one.	10	8.2
I do not think that a transition policy is necessary at this point.	1	.8
I have not given it much thought.	0	-

*Percent obtained from 121 affirmative responses

**Table 2:** Staff involved in the transition services of the respondents’ units

Health professionals in the transition service	Number	Percent
Pediatric rheumatologist	92	76.0
Adult rheumatologist	77	63.6
Nurse	42	34.7
Psychologist	18	14.8
Physiotherapist	18	14.8
Social worker	10	8.2
Occupational therapist	3	2.4
Others^a^	11	9.0

^a^Include: internist, medical assistant, expert patients, orthopedic surgeon, gynecologist

A minority of centers (respondents *n* = 44; 36.4%) use a checklist as part of an individualized transition plan that includes the topics shown in Table 3. The majority of these (40/44; 90.9%), agreed that confirmation of the first contact with adult rheumatologist is critical. Other important topics were the importance of knowledge about disease and treatment issues (39/44; 88.6%), as were the encouraging self-management and independent visits by YP without their parents (35/44; 79.5%).

**Table 3:** Components of existing individualized transition plans

Checklist components^a^	Number	Percent
Self-management, patient is seen without parents	35	79.5
Disease and treatment knowledge	39	88.6
Name of disease	39	88.6
Being able to describe disease course	32	72.7
Signs and symptoms of disease flare	33	75.0
Signs and symptoms that require an urgent consultation	30	68.1
Treatment information	40	90.9
Possible side effects of treatment	36	81.8
Health behavior	31	70.4
Risk behavior	29	65.9
Alcohol use, smoking, illegal drugs use	35	79.5
Nutrition	27	61.3
Sports	31	70.4
Mental health	27	61.3
Sexuality, contraception and sexual health	34	77.2
Future plans	33	75.0
Educational achievements	30	68.1
Vocational readiness	22	50.0
Knowledge of support resources	28	63.6
Mobility, living alone, travel	24	54.5
Medical summary available	34	77.2
Knowledge of differences between pediatric and adult rheumatology care	36	81.8
Knowledge about the health system (health insurance, general practitioner/family doctor, health care specialist)	21	47.7
Transfer readiness	28	63.6
First contact to adult rheumatologist	40	90.9

^a^Percent obtained from 44 affirmative responses on having a checklist

Of note, less than 10% of centers (respondents *n* = 11) use a specific readiness for transfer instrument, of these, five use a self-developed readiness scale. Other instruments used are the Transition Readiness Assessment Questionnaire (TRAQ), TRANSITION Scale, Ready Steady Go [17].

A minority of respondents (18/121; 15%) reported to use or recommend specific resources for YP and their families. These were mainly websites providing information on juvenile rheumatic diseases, but also on transition aspects, such as the PRINTO website [www.printo.it/pediatric-rheumatology] and the website on transition of the Competence Network Patient Training in Adolescence KomPaS e. V. [www.between-kompas.de]). Furthermore, a minority (38/121; 31%) use information technology to facilitate communication with YP; mainly Short Message Service (SMS), web platforms, email, or apps and the remainder tend to communicate by telephone.

Among the rheumatology centers from different models of care, some differences regarding transition care were observed. In comparison to children’s hospitals, University affiliated practices provided somewhat less often transition services (69% vs. 78%), had less often a written transition policy (22% vs. 41%) and used significantly less frequently a checklist as part of individualized transitional care (23% vs. 48%, *p* = 0.011).

**Fig. 1:**
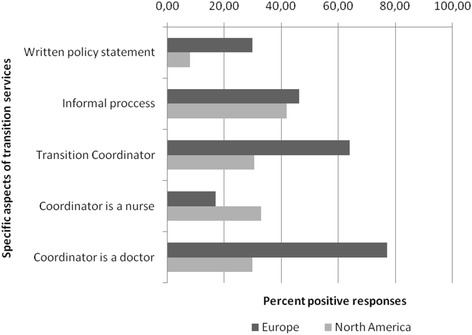
Comparison of transitional care services offered by European and Childhood Arthritis and Rheumatology Research Alliance (CARRA) pediatric rheumatology providers

Figure 1 is a pragmatic attempt to compare the data from our survey and previous published data from North America [12]. Notably the majority of North American respondents (89%) work in a University-affiliated practice in contrast to 49% of their European colleagues (*p* < 0.01; 99% CI: 0.29, 0.49). Current transitional care services in Europe and North America show significant differences; the presence of a designated staff member who coordinates transition (64% in Europe vs 30% in North America; *p* < 0.01; 99% CI: -0.45, −0.22) and the availability of a written transition policy (26% in Europe vs 8% in North America; *p* < 0.001; 99% CI: -0.26, −0.08). Nevertheless, the percent of informal transition process is similar across surveys (46% vs. 42%; non-significant).

## Discussion

This is the first survey to focus on the current practices (rather than solely attitudes or opinions) in transitional care among European pediatric rheumatology providers. As in other chronic diseases that start in childhood, YP with jRMD need specific care during the period of transition from pediatric services to the adult specialty care setting. However, research about current practice and the implementation of transition recommendations are scarce and almost half of the YP with jRMD do not make a successful transfer to adult rheumatology and are, therefore, at increased risk of unfavorable outcomes [5, 18, 19].

Recently, EULAR/PReS recommendations were issued for transitional care throughout Europe [14]. These recommendations were published after the e-survey was conducted and so our data serve as useful benchmark of current practice. The recommendations highlight the need for a written transition policy, and yet less than a third of respondents in our survey indicated that their department had a formal transition program; an ‘informal approach’ was reported to be more common although further information was not given in the survey. It is hoped that the use of the recommendations and a consistent approach to the transition program is more likely to address the structure, assessments of patient readiness and service outcomes. We note that only a small percentage of transition clinics receive funding or reimbursement for the service and it is possible that one barrier to implement more ambitious transition programs may be insufficient resources. Interestingly, the EULAR/PReS recommendations include the need to secure funding for provision of transitional care, in order to avoid some of the major barriers as highlighted by the North American survey and a frequent shortcoming in Europe [14].

The presence of a transition coordinator is another key element of transition and one of the EULAR/PReS recommendations; the aim being to ensure implementation and evaluation of the transition program and improving communication between health professionals, YP and families [8, 14]. Most respondents of the survey have a designated staff member as transition coordinator. However, in contrast to the recommendations, emphasizing the important role of allied health professionals in transition coordination, we note that the designated staff member is often a doctor rather than a nurse, youth worker or other professional. The EULAR/PReS recommendations do not specify whether the coordinator is a doctor or allied health professional but that the individual is clearly identified within the team and has the appropriate skills to facilitate liaison between the pediatric and the adult care teams [14].

Information and education of YP about their disease, treatments and promotion of independent visits to clinic without parents are ideal components of an individualized transition plan. However, it is important to include systematically other important areas of adolescence health, such as risk behavior, substance use, sports, nutrition or sexuality. The HEADSS assessment (Home, Education/Employment, peer group, Activities, Drugs, Sexuality, Suicide/depression) was developed [20] and has since been successfully used to facilitate communication with YP and as guide to cover all topics pertinent to young people in the transition process. However, to address all relevant transition issues, it might be necessary to have a multidisciplinary team (i.e., psychologist to handle mental health or physiotherapist to advice about sports and routine exercises). This approach is clearly recommended in the EULAR/PReS document [14]. Our survey revealed that currently only a minority of centers offers multidisciplinary transitional care services; 35% of the centers have a nurse, 15% a psychologist and 8% a social worker available for addressing transition-relevant issues.

Our survey did not specifically address timing of transfer. However, it should be noted that only 9.5% use a readiness instrument to validate transfer, reflecting the informal approach to transition in the majority of centers. This issue was heavily discussed in the development of the EULAR/PReS recommendations, as some countries had a designated age for transfer in all cases and independently of YP readiness. Ultimately the EULAR/PReS recommendations propose that age per se is not specifically used as the trigger for timing of the transfer. Conversely, age is an important indicator for the initiation of transitional care; namely that the transition process should start as early as possible [14]. The recommendations also propose strategies to facilitate transfer including communication with adult services before actual transfer with relevant information about the YP or shared clinics between pediatric and adult health care professionals. In this way, the timing of transfer could be more flexible, and could be deferred until the disease is stable and/or the transition team consider the patient to be “ready”.

Where possible and with the questions that can be compared, we were able to comment on differences between European and North American respondents. European providers more often do have a transition coordinator (often a doctor) and are more likely to use a written transition policy statement. In both surveys, respondents manifest a strong desire for rheumatology-specific guidelines for transition [12] and for these to be implemented across pediatric, adolescent and adult rheumatology health care settings.

In summary, this survey has demonstrated limitations of existing transition practices and paucity of resources. Nonetheless there is a strong commitment within the rheumatology communities (both adult and pediatric), to improve existing transitional care provision. The EULAR/PReS recommendations are therefore timely and important as a much needed catalyst for change within the rheumatology community.

## Additional file

Additional file 1: Questionnaire on transition practices. (DOC 36 kb)
