# Supplementary material for: Transitional care for rheumatic conditions in Europe: current clinical practice and available resources
Source: Pediatr Rheumatol Online J. 2017 Jun 9;15:49. doi: 10.1186/s12969-017-0179-8 (PMC5466791; doi:10.1186/s12969-017-0179-8)
Supplement: Additional file 1: — Questionnaire on transition practices. (DOC 36 kb) [file 12969_2017_179_MOESM1_ESM.doc]

**Additional file 1**

Questions regarding transition practices:

1. Regarding transition from pediatric to adult care, select one of the below which best describes your practice:

🔿My practice has a written transition policy which we follow most of the time

🔿My practice has a written transition policy, but we do not follow it most of the time.

🔿We do not have a written transition policy but follow a fairly standard, informal
 procedure in transitioning our patients

🔿We are working on developing a transition policy but do not yet have one formalized

🔿We do not have a transition policy but are interested in developing one

🔿I do not think that a transition policy is necessary at this point

🔿I have not given it much thought

1. Is there a designated staff member in your practice who has primary responsibility for coordinating transition process?

🔿Yes 🔿 No

if yes (you do have a designated staff member in your practice), what is his/her role?

🔿Nurse

🔿Office manager/assistant

🔿Social worker

🔿Physician

🔿Other, please specify …………………………

1. Does your institution regularly offer transition services for adolescents with rheumatic diseases?
   🔿Yes 🔿No

   if yes: Who is usually involved in the transition service (please indicate all that apply)

🔿paediatric rheumatologist

🔿adult rheumatologist
🔿nurse
🔿psychologist

🔿 physiotherapist
🔿 social worker
🔿 occupational therapist
🔿 other persons, namely ………………………………..
 please specifiy

Do you receive funding or reimbursement for the service?

🔿Yes 🔿 No

if yes: from whom? 🔿 government

🔿 health insurance company

🔿 others, namely …………………………….
 please specifiy

1. Does your institution use a checklist for providing individualized transitional care?
   🔿Yes 🔿No

   if yes: which aspects are addressed by the checklist (please tick the appropriate)

🔿 self-management, patient is seen without parents

🔿 disease and its treatment 🔿name of disease
🔿being able to describe disease course
🔿 signs and symptoms of disease flare
🔿 signs and symptoms that require an
 urgent consultation
🔿kind of treatment
🔿 possible side effects of treatment

🔿 health behavior 🔿 risk behavior
 🔿alcohol use, smoking, illegal drug use
 🔿 nutrition
 🔿sports
🔿 sexuality, anticonception

🔿 future plans, perspectives
 🔿educational achievements
 🔿vocational preparation and training
🔿 knowledge of available ressources, legal support packages

🔿 mobility, living alone, travel

🔿 having a medical summary
🔿 knowing the differences between pediatric and adult rheumatology
 care
🔿 knowledge about the health system (health insurance, general
 practitioner/family doctor; health care specialist)
🔿 transition readiness
🔿 first contact to adult rheumatologist
🔿 others, namely …………………………………………………………
 please specifiy

1. Does your institution use a specific validated readiness instrument?
   🔿Yes 🔿No
    if yes: please report which one

🔿 Transition Readiness Assessment Questionnaire (TRAQ)
 🔿 Am I ON TRAC for Adult Care

🔿 Transition-Q
🔿 Self-Management Skills Assessment Guide (SMSAG)

🔿 Readiness for Adult CarE in Rheumatology (RACER)
🔿 TRxANSITION Scale

🔿another, namely ………………………………..
 please specifiy

1. Does your institution use or recommend specific resources (e.g. specific websites like <http://www.sickkids.ca/Good2Go/for-health-care-providers/Index.html> or [www.transitioninfonetwork.org.uk](http://www.transitioninfonetwork.org.uk/), brochures)?

🔿Yes 🔿No
 if yes: please report which one: ……………………………………………………..

1. Do you use new media for the communication with adolescent patients?
   🔿Yes 🔿No
    if yes, which one

🔿 SMS

🔿 web platform
🔿 Apps

🔿 social media
🔿 others, namely ………………………………..
 please specifiy

Many thanks for accepting to answer this questionnaire.

Please answer finally these questions.

Please select the one response which best describes your practice

🔿University affiliated practice

🔿General Children´s hospital, government medical centre

🔿Private practice

🔿Other, please specify ……………………………………..

In which country do you practice? ……………………………………
